# The Response of the Alpine Dwarf Shrub *Salix herbacea* to Altered Snowmelt Timing: Lessons from a Multi-Site Transplant Experiment

**DOI:** 10.1371/journal.pone.0122395

**Published:** 2015-04-20

**Authors:** Janosch Sedlacek, Julia A. Wheeler, Andrés J. Cortés, Oliver Bossdorf, Guenter Hoch, Christian Lexer, Sonja Wipf, Sophie Karrenberg, Mark van Kleunen, Christian Rixen

**Affiliations:** 1 Ecology, Department of Biology, University of Konstanz, 78457, Konstanz, Germany; 2 WSL Institute for Snow and Avalanche Research SLF, 7260, Davos, Switzerland; 3 Department of Ecology and Genetics, Uppsala University, 75236, Uppsala, Sweden; 4 Plant Evolutionary Ecology, Institute of Evolution and Ecology, University of Tübingen, 72076, Tübingen, Germany; 5 Institute of Botany, University of Basel, 4056, Basel, Switzerland; 6 Unit of Ecology and Evolution, Department of Biology, University of Fribourg, 1700, Fribourg, Switzerland; INRA - University of Bordeaux, FRANCE

## Abstract

Climate change is altering spring snowmelt patterns in alpine and arctic ecosystems, and these changes may alter plant phenology, growth and reproduction. To predict how alpine plants respond to shifts in snowmelt timing, we need to understand trait plasticity, its effects on growth and reproduction, and the degree to which plants experience a home-site advantage. We tested how the common, long-lived dwarf shrub *Salix herbacea* responded to changing spring snowmelt time by reciprocally transplanting turfs of *S*. *herbacea* between early-exposure ridge and late-exposure snowbed microhabitats. After the transplant, we monitored phenological, morphological and fitness traits, as well as leaf damage, during two growing seasons. *Salix herbacea* leafed out earlier, but had a longer development time and produced smaller leaves on ridges relative to snowbeds. Longer phenological development times and smaller leaves were associated with reduced sexual reproduction on ridges. On snowbeds, larger leaves and intermediate development times were associated with increased clonal reproduction. Clonal and sexual reproduction showed no response to altered snowmelt time. We found no home-site advantage in terms of sexual and clonal reproduction. Leaf damage probability depended on snowmelt and thus exposure period, but had no short-term effect on fitness traits. We conclude that the studied populations of *S*. *herbacea* can respond to shifts in snowmelt by plastic changes in phenology and leaf size, while maintaining levels of clonal and sexual reproduction. The lack of a home-site advantage suggests that *S*. *herbacea* may not be adapted to different microhabitats. The studied populations are thus unlikely to react to climate change by rapid adaptation, but their responses will also not be constrained by small-scale local adaptation. In the short term, snowbed plants may persist due to high stem densities. However, in the long term, reduction in leaf size and flowering, a longer phenological development time and increased exposure to damage may decrease overall performance of *S*. *herbacea* under earlier snowmelt.

## Introduction

Temperature, precipitation and, as a consequence, snowmelt patterns in alpine ecosystems are changing dramatically, with observations of snowmelt acceleration in spring [1]. Model simulations predict further declines of snow-cover duration by 30–80% in the Alps by the end of the century [2]. Since the strongest snow-cover reductions are predicted for spring [2], earlier snowmelt may prolong the growing season above 2000 m asl by up to 60 days [3]. A longer growing season could have strong impacts on plants, as it may alter the timing of phenological development, increase exposure to frost, change moisture availability and alter interactions with pollinators, herbivores and pathogens [3–9]. However, potential impacts have been assessed for only a few alpine species.

To respond to changing snowmelt conditions, alpine shrubs must either track their climate requirements by migrating to sites with suitable microclimates, or persist under the new conditions through phenotypic plasticity or adaptive evolution [10–12]. However, the potential for migration might be limited in species with long generation times [13] and poor dispersal ability [14], and in species growing in fragmented landscapes [15]. Recent studies have also considered how shifts in biotic interactions limit migration potential, including changes in herbivory and pathogen damage [16–21].

If migration potential is limited, the only way plants can respond to change is by adjusting to the new environmental conditions. In the short term, this can be achieved through plasticity, potentially maintaining high clonal and sexual reproductive rates [22]. In the long term, adjustment to climate change can take place through adaptive evolution [23–25]. The latter requires both the presence of genetic variation in relevant traits, and selection acting on these traits. If local adaptation is detected in spite of ongoing gene flow, this suggests strong selective forces [26]. Adaptive evolution, however, might be too slow to keep pace with environmental change, especially in long-lived alpine species [15,25]. Phenotypic plasticity, in contrast, allows plants to rapidly adjust to changing environmental conditions within the lifetime of a species, and thus may play a key role in species responses to climate change, particularly in long-lived species. Phenotypic plasticity has been mainly demonstrated for phenological changes (e.g. in timing of bud burst) in response to warmer temperatures [27,28]. However, phenotypic change could reflect passive plastic responses as a consequence of resource limitation. Such a passive plastic response may be neutral or even maladaptive [29,30], if it does not benefit fitness [31,32]. It is therefore important to understand whether plasticity allows a species to respond to climate change [33], or whether adaptive responses or shorter-distance migration would be needed for regional persistence.

In order to rigorously test how plants respond to snowmelt change through phenotypic plasticity, as well as whether plants experience a home-site advantage, reciprocal transplant experiments are necessary [26,34]. Many previous studies have used such transplant experiments to investigate phenotypic plasticity and local adaptation in alpine plant species across altitudinal gradients [35–37]. However, reciprocal transplant studies explicitly examining the effects of altered snowmelt timing are scarce (but see [38–40]). Further, almost all previous transplant experiments examined short-lived perennial herbs, and experiments with long-lived woody species are extremely rare (but see [38]). However, it is important to understand how long-lived species will respond to changes in snowmelt timing, as they are a dominant functional type in alpine plant communities.

The long-lived, arctic-alpine dwarf shrub *Salix herbacea* typically occurs in late-snowmelt microhabitats but also on wind-exposed mountain ridges [41]. A 3-year observational study in the Swiss Alps, which included a microhabitat and elevation gradient, indicated that most traits of *S*. *herbacea* were affected mainly by snowmelt microhabitat type (Wheeler et al., unpublished). These microhabitats are mainly differentiated by the duration of winter snow cover [42], but also by environmental factors closely linked to or controlled by snowmelt timing, such as temperature, soil moisture, and biotic interactions, which are predicted to change due to climate change. Therefore, this natural microhabitat setup is well-suited to test the effects of climate change-driven shifts in snowmelt timing in a reciprocal transplant experiment.

In order to make predictions about the potential responses of *S*. *herbacea* to changes in snowmelt timing, we must understand to what degree phenotypic plasticity can help to adapt to the new environmental conditions, and whether the species exhibits a home-site advantage in different microhabitats. To this end, we reciprocally transplanted turfs of *S*. *herbacea* between early-exposed ridge and late-exposed snowbed microhabitats. We investigated the effects of both origin and destination microhabitats on morphological, phenological and fitness traits of *S*. *herbacea*, assessed leaf damage, and asked the following questions:

1. Does altered snowmelt timing lead to plastic responses in phenology, leaf size, clonal reproduction and sexual reproduction?
2. Does altered snowmelt timing lead to a change in leaf-damage probability?
3. Does *S*. *herbacea* demonstrate a home-site advantage to local microhabitat snowmelt conditions, suggesting local adaptation?
4. How do phenology, leaf size and damage affect flowering and clonal reproduction within each of the microhabitats?

## Methods

### Study species

*Salix herbacea* L. is a long-lived, clonal, dioecious, prostrate dwarf shrub, occurring in the northern and alpine regions of Eurasia and North America, and the Arctic region [41]. The species produces an extensive ramifying system with branched rhizomes forming flat mats [41]. The aerial branches are woody and usually reach 2–5 cm above the ground surface. *Salix herbacea* is characteristic to a wide range of microhabitat types, from ridge and scree habitats with early exposure from snow in spring and relatively long growing seasons, to snowbeds with long snow cover duration and short growing seasons. Ridges in our study area were dominated by the shrubs *Loiseleuria procumbens* and *Vaccinium uliginosu*m, in addition to the herb *Phyteuma hemisphaericum*. Snowbeds were characterized by the herb *Gnaphalium supinum* and the moss *Polytrichastrum sexangulare*.

### Reciprocal transplant experiment

To test for plastic responses and potential adaptation of *S*. *herbacea* to joint changes in snowmelt patterns and temperature, we established a reciprocal transplant experiment with six pairs of sites. Each pair consisted of an early-exposed ridge site and a nearby late-exposed snowbed site (see S1 Fig and S1 Table, for a description of site characteristics). Our study sites are typical representatives for ridge and snowbed habitats in the Alps ([43], Wheeler et al. unpublished). Snowmelt on ridges occurred on average one month earlier than in snowbeds (mean ± SE Julian day of snowmelt; ridge: 157 ± 3.6, snowbed: 191 ± 3.1) across both years 2012 and 2013, and earlier in 2012 than in 2013 (2012: 170 ± 7.2, 2013: 179 ± 4.4). As a consequence of earlier snow disappearance and exposure to colder spring temperatures, ridges were colder when averaged over the entire growing season compared to snowbeds (mean ± SE of temperature from snowmelt to end of growing season (Julian day 217; ridge: 10.89 ± 0.44°C, snowbed: 11.88 ± 0.50°C). Despite differences in timing of snowmelt, the total growing degrees days per season (sum of growing degree days with a threshold of 5°C, between snowmelt and Julian day 217; GDD) were similar on ridges and in snowbeds (mean ± SE of GDD; ridge: 974.05 ± 106.17°C, snowbed: 922.23 ± 71.35°C, S2 Fig). We focused on snowmelt timing and temperature in our microhabitats, because they are the most prominent aspects of climate change in the Alps, and are generally key regulators of other abiotic and biotic conditions, like soil moisture, nutrient availability, freezing events, and vegetation cover and composition [5,11,42].

The 12 sites were located at similar altitudes (2320–2355 m asl) on a northeast-facing slope near Davos in the eastern Swiss Alps (S1 Fig, S1 Table). The distance between two sites in a pair ranged from 28 to 55 m, and the distance between pairs of sites ranged from 40 to 190 m. Within each site, we haphazardly selected and excavated 14 *S*. *herbacea*-containing soil patches with a diameter of 10 cm and a soil depth of 5 cm. To reduce the probability of sampling the same genotype multiple times [44], we chose patches that were at least 1 m apart and had no visible stem connections. We carefully cut each patch into two halves (turfs), ensuring that each turf contained a minimum of five *S*. *herbacea* stems. We then transplanted one turf back in the same site (“home site”), and transplanted the other turf in the other microhabitat of the site pair (“away site”). Overall, we transplanted 336 turfs (12 sites × 14 patches x 2 turfs). We minimized negative effects of the transplant on plant performance by maintaining large root systems for each turf and by transplanting at the end of the growing season (15th-16th August 2011), after terminal buds had already formed. Since the first two weeks after transplanting were dry, we watered all turfs twice during these two weeks. We used turfs in our reciprocal transplant instead of seeds or seedlings as seed germination in the field has been observed to be extremely low and progress from the seedling stage to sexual maturity is expected to be very slow, as is typical for slow-growing clonal woody shrubs.

### Data collection

At each site, we recorded soil temperature every 2 hours at 5 cm soil depth using in-situ temperature loggers (iButton, Maxim Integrated, San Jose, CA, USA). We used this temperature data, in conjunction with field observations to determine the day of snowmelt for each site as the date when soil temperature rose abruptly from near 0°C (which is the characteristic soil temperature below snow cover in spring).

We assessed a set of phenological, morphological and fitness traits, which have been suggested to be key plant traits for responses to climate change [30]. At transplanting, we counted the number of stems on each turf, and we used this number as a proxy for initial plant size. Over the two growing seasons (2012 and 2013) following transplant, we weekly monitored phenology, proportion of flowering and fruiting stems, and whether any leaves were damaged by herbivores, fungi or gall-forming insects. The development time to each phenophase (leaf expansion, flowering, fruiting) was calculated as the difference from day of snowmelt until onset of the respective phenophase. Development times to each phenophase were strongly correlated with each other (leaf expansion and flowering: *r* = 0.78, *P* <0.001, *n* = 278; leaf expansion and fruiting: *r* = 0.80, *P* <0.001, *n* = 123; flowering and fruiting: *r* = 0.81, *P* <0.001, *n* = 113); so onset of and phenological development time to leaf expansion are discussed as representative phenological variables from this point onwards (see S2 Table for results for flowering and fruiting). At leaf maturity, we assessed individual leaf size (i.e. π x ½ length x ½ width taken from two undamaged, fully-expanded leaves per turf, that were selected haphazardly at the terminal point of two stems) and stem number per turf. Relative changes in stem numbers between 2012 and 2013 were calculated as the ratio of stem number in 2013 to stem number in 2012. So, values larger than one would indicate a net increase, while values smaller than one would indicate a net decrease.

No specific permits were required for the study location and activities, and the field studies did not involve endangered or protected species.

### Statistical Analysis

We used generalized linear models to test whether phenotypic variation was explained by the microhabitat where the turfs were planted (i.e. destination effect, which would indicate phenotypic plasticity), by their site of origin (i.e. origin effect, which would indicate genetic effects or environmental carry-over effects), or an interaction of both (i.e. destination x origin effect, which could indicate a home-site advantage or disadvantage).

The response variables leaf size, and onset of and phenological development time to leaf expansion were analyzed with a Gaussian error distribution. Leaf size was log-transformed to achieve normality and homoscedasticity of the residuals. For stem number, we used a Poisson error distribution, and for the proportion of flowering and fruiting stems and the presence of leaf damage, we used a binomial error distribution. For each response variable, the models included microhabitat of origin (ridge vs. snowbed), microhabitat of destination (ridge vs. snowbed), year (2012 and 2013) and their interactions as fixed effects. To associate the two measurements per turf with their corresponding years, we also included year as a fixed effect in all models. We considered turf nested within patch, nested within destination site, nested within pair as random effects, to account for non-independence of measurements repeated on the same turfs in different years, and non-independence of patches from the same sites within pairs. Proportions of flowering stems were analyzed only for 2013, because flower buds in 2012 were pre-formed in the year before the transplant. Fruit set was excluded from the analysis, due to the low number of fruiting plants in 2013 (N<25 turfs). In these analyses, with only a single measurement per turf, we excluded year as a fixed and turf as a random effect. To test whether the proportion of flowering stems depended on the sex of the plant, we added sex as an additional fixed factor, as well as interactions between sex and microhabitat of origin and microhabitat of destination. We also ran separate models for female and male plants. To account for differences in stem number at transplant, we included initial stem number (from 2011) as a covariate in the model for stem number (see also S1 Equation for the R code of the models). We used log-likelihood-ratio tests to determine the overall significance of main effects and interactions [45]. We assessed the significance of each three-way interactions by comparing the full model to the model from which the respective three-way interaction was removed. We assessed the significance of each two-way interaction by comparing the model without three-way interactions to the model from which the respective two-way interaction was removed. We assessed the significance of each main factor by comparing the model without interactions to the model from which the respective main factor was removed. The significance of the covariate stem number was assessed by comparing the model without main factors to the model from which the covariate was removed. For each of these model comparisons (see S3 Table for an overview), we also report the changes in AIC values (delta AIC values). We also calculated marginal *R* ^2^ (proportion of variance explained by the fixed effects only) and conditional *R* ^2^ (proportion of variance explained by both the fixed and random factors) as a measure of goodness-of-fit of each model [46].

To test whether phenology and leaf size were under selection in the ridge and snowbed sites, and to determine whether leaf damage impacted clonal and sexual reproduction, we used selection-gradient analyses [47]. We analyzed turfs from the two origins jointly within each microhabitat type, which increased the amount of phenotypic variation in the analysis and thus increased the power for estimating the shape of the fitness surface. As proxies for sexual and clonal reproduction, we used flowering probability and the relative change in stem number between 2012 and 2013, which includes both the growth of new stems and stem die-off. Using multiple regression mixed models, we regressed the relative change in stem number and flowering probability on linear and quadratic terms of leaf size, measured in the previous year, phenological development time (duration from snow disappearance to leaf expansion) and damage probability, for ridge and snowbed destinations separately. All traits were standardized on the site level. To account for differences in selection gradients between turfs from different origins, we included origin and its interactions with the linear and quadratic terms of each trait. For the relative change in stem number, we used a Gaussian error distribution, and for flowering probability, we used a binomial error distribution. Because flowering probability is influenced by stem number, we included stem number as an additional explanatory variable. We included site as random effect, to account for non-independence of turfs measured in the same site. Starting from the full model, nested models were compared using a likelihood ratio test (LRT; Chi-square test statistic) by first dropping each non-significant interaction term, followed by the non-significant quadratic terms [45]. We used the reduced optimal model to estimate linear (β) and quadratic (γ, i.e. 2 x quadratic coefficient) selection gradients and their confidence intervals (bootstrapped with 1000 iterations).

All statistical analyses were performed using linear and generalized linear mixed models as implemented in the *lme4* package [48] in R version 2.0.3 [49].

## Results

### Clonal and sexual reproduction

**Fig 1:**
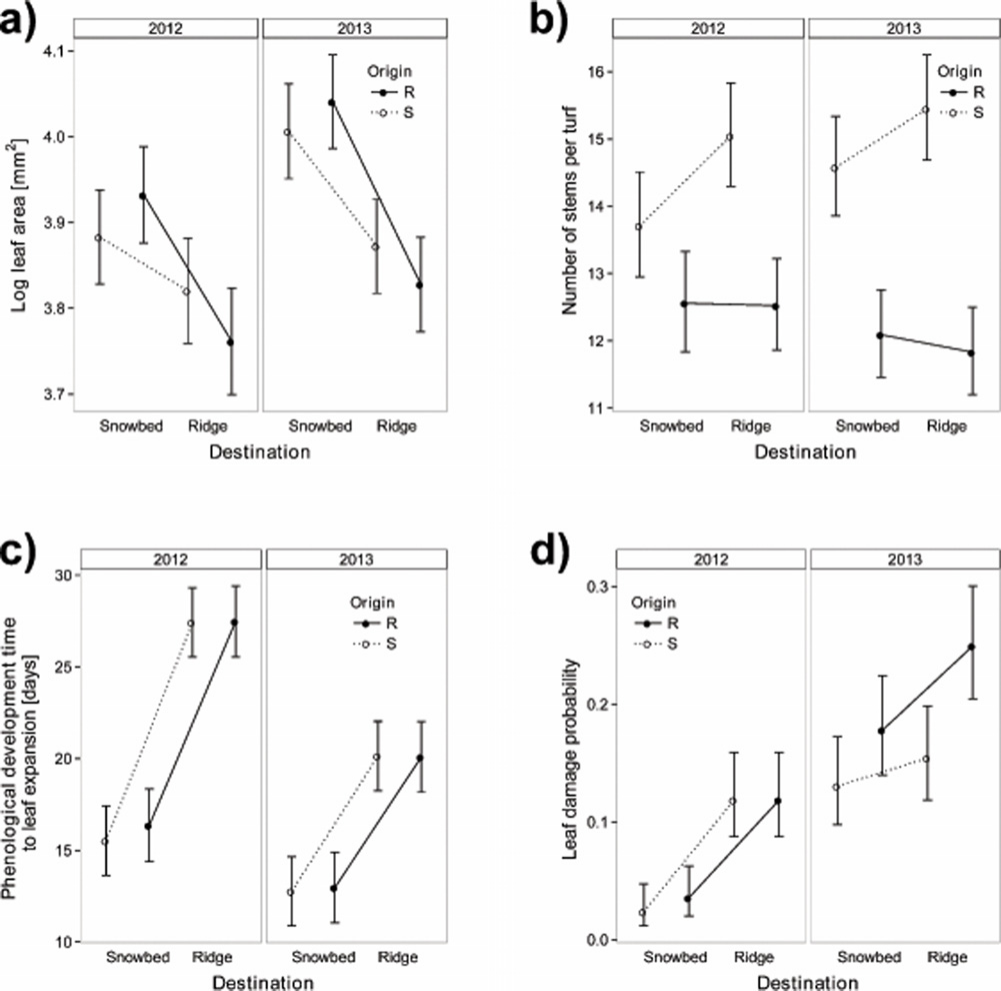
Leaf size (a), stem number (b), phenological development time (time from snowmelt to leaf expansion) (c), and leaf damage probability (d) of *Salix herbacea* turfs, reciprocally transplanted in 2011 between late exposed snowbed and early exposed ridge microhabitat sites in an alpine tundra site near Flüelapass, Switzerland. Turfs originating from ridges (R) are marked with solid lines and filled circles, turfs originating from snowbeds (S) with dashed lines and open circles. Errorbars show standard errors.

The number of stems was significantly higher for *S*. *herbacea* turfs originating from snowbeds than for those from ridges (Table 1). The difference in number of stems increased over time depending on turf origin (Table 1, Fig 1b). Although turfs originating from snowbeds showed higher stem numbers on ridges, whereas turfs from ridges did not differ in stem numbers between the destinations, the destination-by-origin interaction was not significant. Overall, these results indicate that initial differences in stem number between origins did not disappear but even became larger during the course of the study, and that plants from snowbeds did not see significant reductions in stem number when transplanted to ridges.

**Table 1:** The effects of destination, origin, year and sex respectively, and their interactions, on the leaf size, stem number, phenology (duration from snowmelt to leaf expansion phenophase), leaf damage, ratio of flowering and ratio of fruiting stems of reciprocally transplanted *Salix herbacea* turfs.

	Leaf size			Stem number			Onset of leaf expansion			Development time to leaf expansion			Leaf damage			Flowering stem ratio		
Source of Variation	dAIC	χ^2^	*P*	dAIC	χ^2^	*P*	dAIC	χ^2^	*P*	dAIC	χ^2^	*P*	dAIC	χ^2^	*P*	dAIC	χ^2^	*P*
Stem number 2011	-	-	-	251.52	253.52	**<0.001**	-	-	-	-	-	-	-	-	-	-	-	-
Year	2.16	14.16	**<0.001**	-1.91	0.09	0.767	39.31	41.30	**<0.001**	49.38	51.38	**<0.001**	15.20	17.20	**<0.001**	-	-	-
Sex	-	-	**-**	-	-	-	-	-	-	-	-	-	-	-	-	-0.71	1.29	0.257
Destination	2.70	4.70	**0.030**	-1.60	0.40	0.526	23.33	25.33	**<0.001**	7.75	9.75	**0.001**	3.47	5.47	**0.019**	0.94	2.94	0.087
Origin	-2.00	0.00	0.966	11.90	13.90	**<0.001**	0.13	2.13	0.144	-1.83	0.17	0.682	0.37	2.37	0.123	4.35	6.35	**0.012**
Destination:Year	-0.67	1.33	0.250	-1.55	0.45	0.502	99.98	101.99	**<0.001**	7.33	9.33	**0.002**	2.27	4.27	**0.039**	-	-	-
Destination:Sex	-	-	-	-	-	-	-	-	-	-	-	-	-	-	-	1.52	3.52	0.061
Origin:Year	-2.00	0.00	0.966	2.35	4.35	**0.037**	0.61	2.61	0.107	-1.93	0.07	0.793	-1.35	0.65	0.421	-	-	-
Origin:Sex	-	-	-	-	-	**-**	-	-	-	-	-	-	-	-	-	1.01	3.01	0.083
Origin:Destination	-0.38	1.62	0.203	-1.16	0.84	0.359	-1.69	0.31	0.578	-1.87	0.13	0.715	-1.97	0.02	0.882	-0.04	1.96	0.162
Origin:Destination:Year	-1.91	0.09	0.763	-1.97	0.04	0.852	-1.61	0.39	0.531	-1.96	0.04	0.849	-1.70	0.30	0.584	-	-	-
Origin:Destination:Sex	-	-	-	-	-	-	-	-	-	-	-	-	-	-	-	-1.39	0.62	0.433
**Random effects**	**-**	SD		-	SD		-	SD		-	SD		-	SD		-	SD	
Turf/Patch/Plot/Site	-	0.1552		-	0.3365		-	<0.0001		-	<0.0001		-	<0.0001		-	0.2595	
Patch/Plot/Site	-	0.1880		-	0.1511		-	<0.0001		-	<0.0001		-	0.0009		-	0.5205	
Plot/Site	-	0.1018		-	0.0295		-	5.1790		-	4.4440		-	0.1047		-	0.0028	
Site	-	<0.0001		-	0.0002		-	<0.0001		-	<0.0001		-	<0.0001		-	0.0032	
Residual	-	0.2823		-	-		-	5.2800		-	7.1030		-	-		-	-	
**marginal *R*** ^**2**^ **; conditional *R*** ^**2**^	**-**	0.05	0.49	-	0.52	0.84	-	0.78	0.88	-	0.31	0.48	-	0.18	0.18	-	0.08	0.17
**AIC (full model)**	493.3	-	-	3802.9	-	-	2573.8	-	-	2851.9	-	-	502.3	-	-	285.9	-	-

Ratio of flowering and fruiting stems was measured only in 2013, so year was excluded from these models. Initial stem number of 2011 was used as a covariate in the model for stem number (see Methods for details). Log-likelihood ratio tests were used to obtain χ^2^ test statistic. We also report delta AIC (dAIC) values for the model comparisons (see Methods and S3 Table for details on the comparisons).

**Fig 2:**
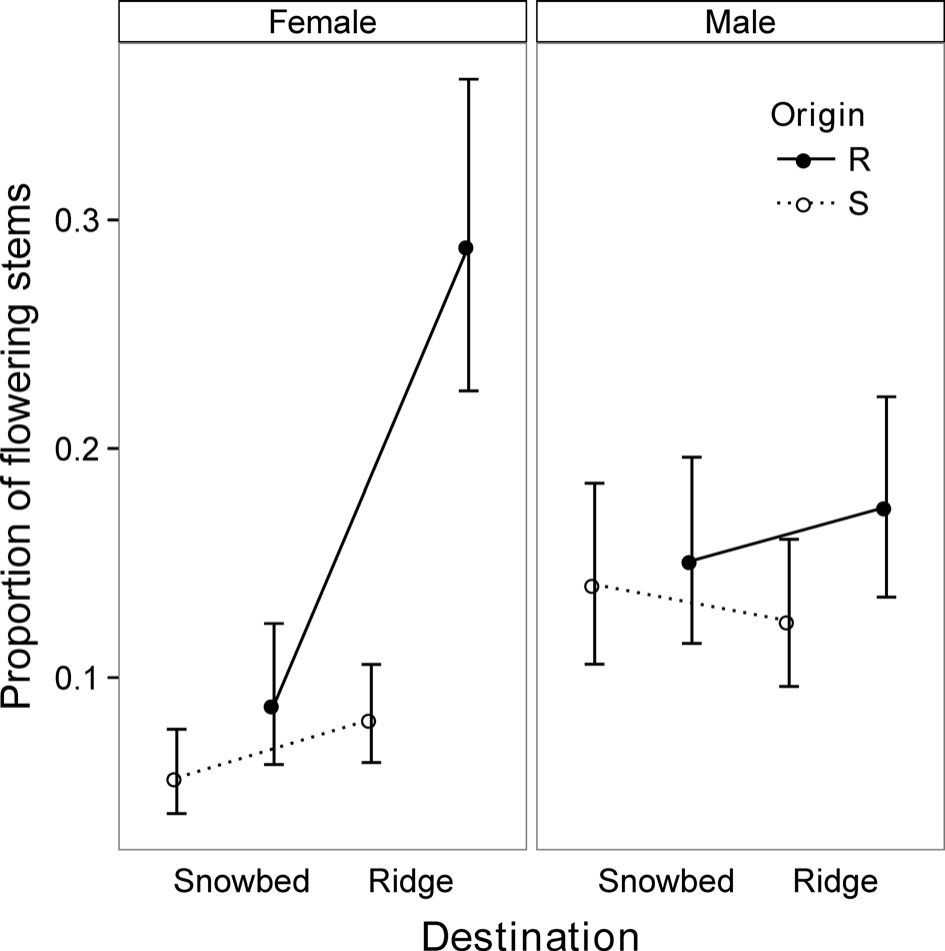
Proportion of flowering female and male stems of *Salix herbacea* turfs, reciprocally transplanted in 2011 between late exposed snowbed and early exposed ridge microhabitat sites in an alpine tundra site near Flüelapass, Switzerland. Turfs originating from ridges (R) are marked with solid lines and filled circles, turfs originating from snowbeds (S) with dashed lines and open circles. Error bars show standard errors.

Turfs originating from ridges had on average a greater proportion of flowering stems than turfs from snowbeds (Table 1, Fig 2), and ridge and snowbed turfs produced a similar absolute number of flowering stems (ridge turfs: 2.60 ± 0.38, snowbed turfs: 2.55 ± 0.37). The origin effect was evident for both sexes, and present in both microhabitats (Table 1). Further, there was a non-significant, but close to significant, interaction effect between sex and destination (*P* = 0.061, Table 1), with female plants producing a greater proportion of flowering stems on ridges relative to snowbeds, whereas males flowered similarly in both microhabitats. When we analyzed female plants separately, the destination effect was significant (d.f. = 1, χ^2^ = 5.961, *P* = 0.014). This destination effect was stronger for ridge turfs, which performed better at their home site (ridge) compared to their away site (snowbed), but this was not the case for snowbed turfs (Fig 2, Female). However, this pattern did not translate into a significant destination-by-origin interaction.

### Phenology

**Fig 3:**
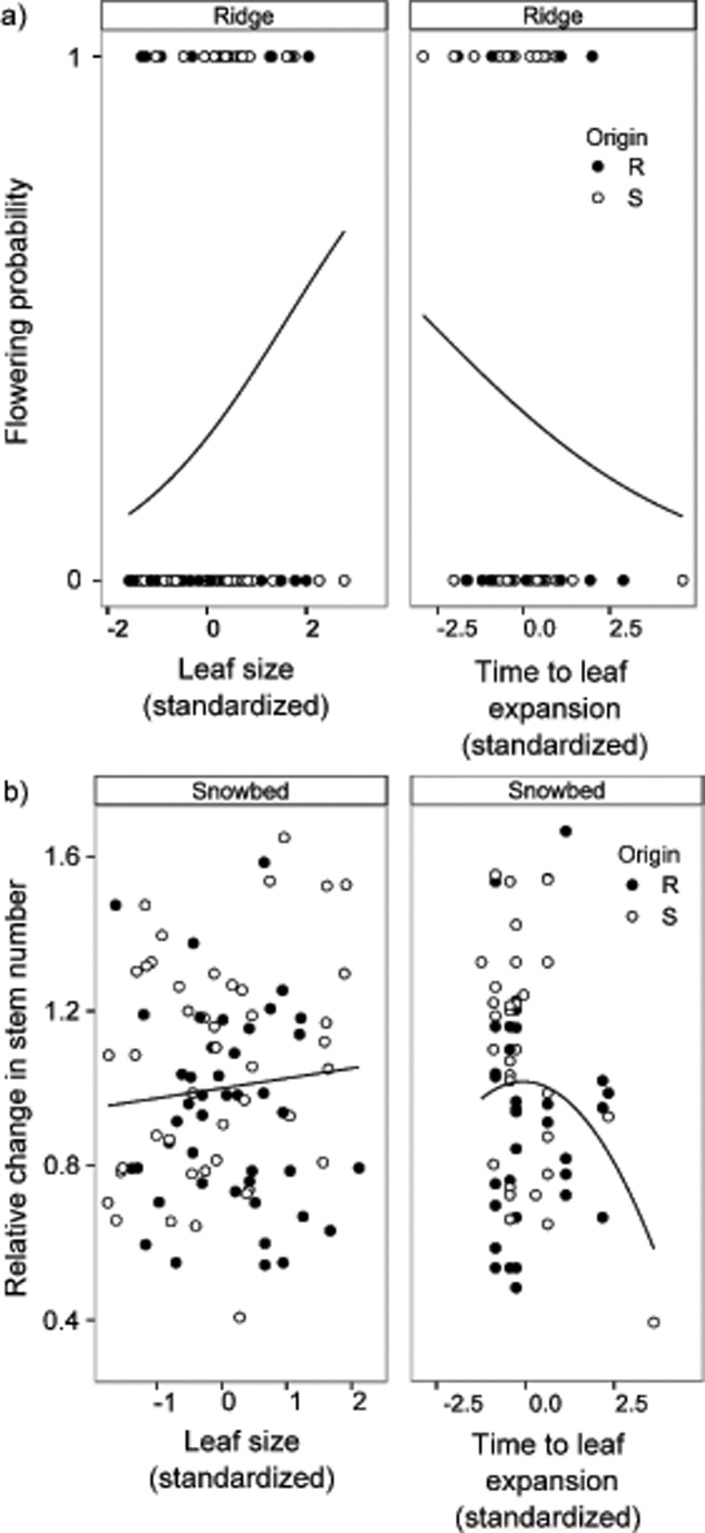
Significant linear and quadratic selection gradients using a) a proxy for sexual reproductive fitness (flowering probability) and b) a proxy for clonal reproductive fitness (relative change in stem number from 2012 to 2013) of *Salix herbacea* turfs growing in ridge and snowbed microhabitat sites (panels). Turfs originating from ridges (R) are marked with solid lines and filled circles, turfs originating from snowbeds (S) with dashed lines and open circles.

Onset of leaf expansion and time from disappearance of snow to leaf expansion were significantly influenced by microhabitat of destination, with turfs in snowbeds expanding their leaves later and requiring less time for this after snowmelt relative to turfs on ridges (Table 1, Fig 1c). Both onset of and phenological development time to leaf expansion were not influenced by origin, but both were influenced by year and a year x destination interaction, due to a very early snowmelt in 2012. As other phenological stages were strongly correlated (see Methods), this suggests that both onset of the phenophase and the phenological development time are controlled by the destination environment in a similar way for plants originating from snowbeds and ridges, and thus respond plastically to environmental differences between the microhabitats. Selection gradient analysis showed that the relative change in stem number as a measure for clonal reproductive fitness, was highest with intermediate development time to leaf expansion in snowbed sites (Fig 3b, Table 2). The flowering probability increased with shorter development time to leaf expansion at ridge sites (Fig 3a, Table 2).

**Table 2:** Estimates, bootstrapped confidence intervals and likelihood ratio test statistics of the reduced optimal models (see Methods for details) for multivariate linear (β) and quadratic (γ) selection gradients at ridge and snowbed sites, using a proxy for sexual reproductive fitness (flowering probability) and a proxy for clonal reproductive fitness (relative stem ratio between 2013 and 2012).

Fitness proxy and micro-habitat	Trait	Linear selection gradient					Quadratic selection gradient					R^2^ reduced optimal model
		β	lowCI, upCI	dAIC	χ^2^	*P*	γ	lowCI, upCI	dAIC	χ^2^	*P*	
**Flowering probability**												
	Leaf size	0.296	-0.47, 0.52	-0.4	1.59	0.208	-	-	-	-		
**Snowbed**	Development time to leaf expansion	-0.071	-0.54, 0.55	-2.0	0.01	0.912	-	-	-	-	-	0.30
	Leaf damage probability	-	-	-	-	-	-	-	-	-	-	
	Leaf size	0.591	0.09, 1.27	3.2	5.21	**0.022**	-	-	-	-	-	
**Ridge**	Development time to leaf expansion	-0.697	-1.64, -0.14	3.7	5.75	**0.016**	-	-	-	-	-	0.44
	Leaf damage probability	0.484	-1.18, 1.97	-1.6	0.47	0.495	-	-	-	-	-	
**Relative change in stem number**												
	Leaf size	0.090	0.02, 0.16	5.0	7.05	**0.008**	-	-	-	-	-	
**Snowbed**	Development time to leaf expansion	0.053	-0.04, 0.15	-0.9	1.17	0.279	-0.142	-0.26, -0.02	3.9	5.88	**0.015**	0.27
	Leaf damage probability	0.136	-0.14, 0.39	-1.0	1.05	0.306	-	-	-	-	-	
	Leaf size	0.030	-0.03, 0.09	-1.2	0.81	0.367	-	-	-	-	-	
**Ridge**	Development time to leaf expansion	0.031	-0.03, 0.1	-1.2	0.83	0.361	-	-	-	-	-	0.07
	Leaf damage probability	0.051	-0.14, 0.23	-1.7	0.32	0.572	-	-	-	-	-	

Note that a quadratic term was kept only in the reduced optimal model for the analysis using clonal fitness at snowbed sites. We also report delta AIC (dAIC) values for the comparisons between the model from which the respective parameter has been removed and the reduced optimal model.

### Leaf size

Leaf size strongly differed between microhabitats of destination and between years. Shrubs produced larger leaves in snowbeds than on ridges (Table 1). This destination-site effect was consistent across both years, but overall leaves were larger in 2013 than in 2012 (Table 1). There were no significant interactions among origin, destination and year. These results suggest that leaf size is a highly plastic trait, responding to environmental differences between the microhabitats and between years. Selection gradient analyses indicated that the relative change in stem number from 2012 to 2013 increased with increasing leaf size in 2012 in snowbed sites, indicating that a plastic increase in leaf size as a response to later snowmelt would benefit clonal reproductive fitness (Fig 3b, Table 2). Flowering probability decreased with decreasing leaf size on ridges, indicating that a plastic reduction in leaf size as a response to earlier snowmelt would not benefit sexual reproductive fitness (Fig 3a, Table 2).

### Leaf damage

The probability of leaf damage by herbivores and pathogens was significantly affected by destination, and the magnitude of this effect changed between years (i.e. there was a significant destination by year interaction, Table 1). Damage probability was higher on ridges than in snowbeds (Table 1, Fig 1d), and while this effect was consistent across years, it was overall much stronger in 2012 than in 2013. Origin of turfs did not affect damage probability, suggesting that all shrubs were equally vulnerable when exposed to damage agents on ridges. Selection gradient analysis showed that damage did not affect clonal or sexual reproduction in either of the microhabitats in the short-term (Table 2).

## Discussion

In our study, *S*. *herbacea* demonstrated evidence of both trait plasticity and microhabitat origin effects. *Salix herbacea* turfs that were transplanted between early and late snowmelt microhabitats exhibited a rapid plastic response to the change in microhabitat for both phenological development and leaf size. In contrast, flower production and stem number were only affected by the microhabitat of origin, at least during the two years of our study. We found no evidence of a home-site advantage of *S*. *herbacea* for any of the measured fitness traits. Reduced leaf size and longer phenological development time was associated with a decrease in flowering probability on ridges. Plants were more likely to incur leaf damage by herbivores and pathogens on ridges than in snowbeds. This suggests that although the studied populations of *S*. *herbacea* can respond plastically to earlier snowmelt by adjusting phenological and morphological traits, exposure to leaf damage agents becomes more likely. Further, passive plastic reduction in leaf size and an increase in phenological development time with earlier snowmelt may lead to reduced flowering, thereby lowering fitness.

### Microhabitat origin and destination effects

Phenology responded strongly to changes in snowmelt timing. Turfs on ridges started earlier but developed more slowly to leaf expansion, flowering and fruiting, while turfs in snowbeds started later but developed faster. Phenology in dwarf shrubs is often closely linked to snowmelt timing, with accelerated snowmelt leading to earlier phenological start in many arctic and alpine species [5,50]. In a field survey on *S*. *herbacea*, Wheeler et al. (unpublished) found similar growing-degrees days (GDD) accumulation thresholds required for starting leaf expansion and flowering along both elevation and snowmelt-timing gradients. Similarly, many arctic and alpine plants must reach GDD temperature accumulation thresholds in order to move to the next phenophase [51–55]. Thus, the mechanism driving the starting time and progression of *S*. *herbacea* phenology is likely temperature, controlled by GDD accumulation beginning immediately after snowmelt. In our transplant sites, slower phenological development on ridges is then a response to colder temperatures in the early growing season.

Leaf size was highly plastic, and increased when turfs were transplanted to snowbeds compared to when they were on ridges. Similarly, Walker et al. found larger leaves produced under later snowmelt conditions in other alpine species [56] (but see [39]). The mechanisms driving larger leaf size in snowbeds are potentially higher temperatures, nutrient and/or water availabilities. Hudson et al. showed that leaf size increased with warming in *Salix arctica*, another prostrate willow, and that this effect remained consistent through a long-term warming experiment [57]. In our study area, later snowmelt timing resulted in warmer growing season temperatures, and nearby alpine sites showed both more bioavailable phosphorus and higher water availability soon after snowmelt [9], both factors which could potentially drive larger leaf sizes in snowbeds. In ridge microhabitats, a reduction in leaf size could have been driven by competition with taller alpine shrubs; however, a concurrent neighbor removal study examining interactions between *S*. *herbacea* and the surrounding vegetation community in the same research area showed that *S*. *herbacea* produced larger leaves in ridge microhabitats when growing in association with taller neighbours [58]. The rapid increase in leaf size under later snowmelt conditions suggests that shrubs can maximize photo-assimilation during the shorter growing seasons [56]. However, we could not detect an increase in clonal and sexual reproduction with bigger leaf size under later snowmelt conditions. In contrast, selection acted on leaf size with earlier snowmelt, since there was a decrease in flowering with smaller leaves on ridges; the fitness costs of these results are discussed below.

Female plants had a lower proportion of flowering stems in snowbeds compared to ridges, and this response was particularly strong for ridge plants. When plants are exposed to the stressful environmental conditions on ridges, they might increase their allocation to sexual reproduction; such shifts in allocation to sexual reproduction are a common response to stressful environments [59]. This increased maternal investment might enable *S*. *herbacea* to disperse to habitats with more suitable environmental conditions. The proportion of flowering stems of male plants and stem number generally did not change in response to the destination microhabitat, but demonstrated strong effects of the origin microhabitat, despite corrections for initial stem number. Although these origin effects on flowering and stem numbers could indicate genetic differentiation, we must interpret them cautiously as there are several alternative explanations. The observed origin effects may be explained by an experimental period too short to detect differences in slow-responding *S*. *herbacea* traits (e.g. clonal growth), and might thus reflect maternal carry-over effects [60]. We accounted for potential carry-over effects due to differences in plant size by including the initial number of stems as a covariate in the analyses. However, these effects might also have been influenced by a correlation with the age of the plant, which is impossible to determine in the field. Another confounding factor could be that the transplanted soil might have contained soil biota and nutrients. However, transplanting with soil and soil biota might have also reduced the number of factors confounded with the shift in snowmelt timing, our main variable of interest. However, at minimum, we speculate that these slow responses in clonal and sexual reproduction might provide a buffer when snowmelt conditions change. Snowbed shrubs, with their high stem density, may prove particularly resistant, due to their potentially high flower production capacity and resource storage.

### Limited evidence for a home site advantage

Highly structured alpine landscapes with steep environmental gradients pose divergent selection pressures on, and lead to restricted gene flow between plant populations, which can promote local adaptation. A home-site advantage or other evidence for local adaptation has been found in many arctic and alpine plants [32,36–38], but see [35,61]. For our study species, ridge and snowbed microhabitats are often extensively temporally isolated by snowmelt timing [4,62], which results in significant temporal separation in flowering times [62]. Despite this phenological isolation between early and late snowbed sites, we did not find any indications for a home-site advantage to microhabitats, characterized by snowmelt timing, using either a sexual reproductive trait (proportion of flowering stems) or a clonal reproductive trait (the relative change in stem number) as fitness traits. This lack of a home-site advantage could have arisen through either carry-over effects or a lack of local adaptation, possibly due to high gene flow [62], and particularly through the mechanism of high seed deposition in snowbed microhabitats, leading to little differentiation between ridge and snowbed plants [62]. In a greenhouse experiment, where we used *S*. *herbacea* seeds and soil from the same study area, we found no indication for local adaptation to soil biota in ridge and snowbed microhabitats[21]. The lack of a home-site advantage suggests that there is no evidence for small-scale adaptive divergence within *S*. *herbacea* populations. This might be beneficial, if climate change leads to an advance in snowmelt timing and/or forces the dwarf shrub to migrate to new snowmelt microhabitats within the current range. However, a lack of small-scale adaptive divergence could also suggest limitations in the evolutionary potential of *S*. *herbacea*.

### Fitness consequences under an early snowmelt scenario

In alpine regions climate change is predicted to drastically advance snowmelt timing and increase temperatures, together with changes in other abiotic and biotic factors, like soil moisture, nutrient availability and biotic interactions [3]. These combined effects may strongly affect plant phenology, morphology and consequently fitness. In our study, we found that flowering probability decreased with a longer phenological development time on ridges. Under accelerated spring snowmelt conditions, a longer phenological development time might increase exposure to episodic freezing damage early in the season, when tissues are in the active growing stages and thus more vulnerable to damage. Since flower buds and flowers are especially vulnerable to freezing this might consequently lead to a reduction in sexual reproductive fitness [6,63]. Furthermore, Stinson showed negative selection on longer flowering development time, likely because of late-season declines of soil moisture in early snowmelt sites [39]. In contrast plant fitness might also benefit from a longer snow-free period, as there is more time for growth and resource allocation [64,65] though many dwarf shrubs show no link between advanced phenology and increased sexual reproduction [5]. Further, we found that the highest values of clonal reproductive fitness were achieved by plants with intermediate values of phenological development time in snowbed microhabitats (Fig 3b). This suggests that clonal growth is reduced in snowbed plants which break winter dormancy first and develop rapidly since they may consequently suffer late spring freezing stress, and also in plants which break later and develop too slowly, so that the remaining growing season might be too short to maximize allocations to growth.

Plant fitness is often demonstrated to benefit from relatively large leaves, by maximizing photosynthetic gains under cool, moist and shaded conditions [66]. In contrast, there might be a trade-off, selecting smaller leaves under hot, dry, high light and low nutrient conditions [67]. Indeed, we found *S*. *herbacea* produced smaller leaves when they were on the drier, more exposed ridges than when they were in snowbeds. Nevertheless, we found directional selection for larger leaves on ridges (i.e. plants with larger leaves were more likely to produce flowers). This suggests that the observed plastic reduction in leaf size under early snowmelt may not be adaptive [29]. Since, as previously discussed, competition does not appear to reduce leaf sizes on early snowmelt sites, the reduction in leaf size most likely reflects a passive plastic reduction as a consequence of lower resource availability on ridges. This passive reduction in leaf size might potentially lead to a reduction in flowering under early snowmelt conditions.

Under early snowmelt, the damage probability increased significantly. Although studies examining the frequency and severity of insect herbivore damage under earlier snowmelt are uncommon, increasing damage by herbivores and pathogens has been found in a long term warming experiment of alpine meadow plants by [7]. This trend is likely driven by a higher abundance of herbivores and pathogens and the prolonged exposure time of early snowmelt sites, which allow for greater developmental periods for growth and reproduction of insect herbivores. Gerdol *et al*. demonstrated that leaf damage led to a decrease in plant fitness under earlier snowmelt conditions [68]. In this study, we found no evidence that higher damage resulted in a lower flowering probability or a decline in stem numbers. However, a larger multi-year study showed reductions on female flowering probability of *S*. *herbacea* in the year following herbivory and fungal damage (Wheeler et al. unpublished). Thus, future studies should investigate male and female reproductive success in more detail, and assess changes in stem numbers over more years.

Despite responding to a change in snowmelt timing through plastic adjustment, plants might also be able to tolerate the new conditions through traits that increase their resistance. Our study suggests that plants from snowbeds can maintain a large size (stem numbers) in the short term (2 years) following a significant change in snowmelt timing. We suggest that this resistance is provided by increased resource storage due to high initial stem numbers. However, over the long term many alpine species have shown lagging population dynamics with currently occupied habitats becoming unsuitable, which leads to an extinction debt [69]. We speculate that many microhabitats may become unsuitable for *S*.*herbacea* due to earlier snowmelt conditions.

## Conclusions

In the studied populations of the alpine dwarf shrub *S*. *herbacea*, phenology and leaf size were strongly responsive to environmental changes triggered by shifts in snowmelt timing. Leaf size and phenological development time had a significant influence on plant fitness traits. Leaf damage probability was controlled by the environment, but appeared to have no fitness consequences in the short term. None of the *S*. *herbacea* turfs from early- or late snowmelt microhabitats demonstrated a home-site advantage, suggesting that the potential of *S*. *herbacea* to adapt to new snowmelt conditions might be limited. Sexual and clonal reproduction did not respond rapidly to snowmelt change; thus under early snowmelt conditions snowbed plants may still perform well in the short term, due to their high stem density relative to individuals from ridges. However, with accelerated spring snowmelt in the long term, exposure to damages, reductions in leaf size and a longer phenological development time could lead to reduced flowering and possibly to population decline in the studied populations of *S*. *herbacea*.

## Supporting Information

S1 Equation R-code for models. R-code for models for the response variable y (i.e. a) leaf size, and onset of and phenological development time to leaf expansion, b) stem number, c) proportion of flowering and fruiting stems and d) presence of leaf damage) used to test whether phenotypic variation was explained by a destination effect, which would indicate phenotypic plasticity, an origin effect, which would indicate genetic effects or environmental carry-over effects, or an interaction of both (i.e. destination x origin effect, which could indicate a home-site advantage or disadvantage).
(DOCX)

S1 Fig Map of the study area. Map of the study area near Davos (Switzerland), and locations of the six pairs of study sites (1–6), each consisting of one early exposed ridge microhabitat (filled circle) and one late exposed snowbed microhabitat (open circle).
(DOCX)

S2 Fig Accumulated growing degree days. Accumulated growing degree days (GDD, 5°C baseline) on ridge- (red) and snowbed sites (blue) in 2012 and 2013. The data were collected on six ridge and six snowbed microhabitat sites used in a reciprocal transplant study established in 2011 in an alpine site near Flüelapass, Switzerland.
(DOCX)

S1 Table Study site characteristics. Geographical coordinates, mean snowmelt date, mean growing season temperature and accumulated growing degree days (GDD, 5°C baseline) over the growing season of the 6 ridge and 6 snowbed microhabitat sites used in a reciprocal transplant study established in 2011 in an alpine site near Flüelapass, Switzerland.
(DOCX)

S2 Table Results for flowering and fruiting. The effects of destination, origin, year and their interactions, on phenology (onset of leaf expansion, onset of flowering, phenological development time to flowering, phenological development time to fruiting) of reciprocally transplanted *Salix herbacea* turfs. Ratio of flowering and fruiting stems was measured only in 2013, so year was excluded from these models. Initial stem number of 2011 was used as a covariate in the model for stem number (see Methods for details). Log-likelihood ratio tests were used to obtain χ^2^ test statistic.
(DOCX)

S3 Table Model comparisons used for the results presented in Table 1 of the main manuscript. The formulas of the full model (1) and the submodels (2–9 or 2–11) are given in the lower part of the table.
(DOCX)
