# Supplementary material for: The Response of the Alpine Dwarf Shrub Salix herbacea to Altered Snowmelt Timing: Lessons from a Multi-Site Transplant Experiment
Source: PLoS One. 2015 Apr 20;10(4):e0122395. doi: 10.1371/journal.pone.0122395 (PMC4403918; doi:10.1371/journal.pone.0122395)
Supplement: S1 Equation — R-code for models for the response variable y (i.e. a) leaf size, and onset of and phenological development time to leaf expansion, b) stem number, c) proportion of flowering and fruiting stems and d) presence of leaf damage) used to test whether phenotypic variation was explained by a destination effect, which would indicate phenotypic plasticity, an origin effect, which would indicate genetic effects or environmental carry-over effects, or an interaction of both (i.e. destination x origin effect, which could indicate a home-site advantage or disadvantage). (DOCX) [file pone.0122395.s001.docx]

**S1 Equation. R-code for models.** R-code for models for the response variable y (i.e. a) leaf size, and onset of and phenological development time to leaf expansion, b) stem number, c) proportion of flowering and fruiting stems and d) presence of leaf damage) used to test whether phenotypic variation was explained by a destination effect, which would indicate phenotypic plasticity, an origin effect, which would indicate genetic effects or environmental carry-over effects, or an interaction of both (i.e. destination x origin effect, which could indicate a home-site advantage or disadvantage).

a) glmer(y~origin*destination*year+(1|pair/site/patch/turf), family="gaussian"

b) glmer(y~Nstems2011+origin*destination*year+(1|pair/site/patch/turf),family="poisson")

c) glmer(y~origin*destination*sex+(1|pair/site/patch),family="binomial")

d) glmer(y~origin*destination*year+(1|pair/site/patch/turf),family="binomial")
