# Supplementary material for: The Response of the Alpine Dwarf Shrub Salix herbacea to Altered Snowmelt Timing: Lessons from a Multi-Site Transplant Experiment
Source: PLoS One. 2015 Apr 20;10(4):e0122395. doi: 10.1371/journal.pone.0122395 (PMC4403918; doi:10.1371/journal.pone.0122395)

**S1 Fig. Map of the study area.** Map of the study area near Davos (Switzerland), and locations of the six pairs of study sites (1-6), each consisting of one early exposed ridge microhabitat (filled circle) and one late exposed snowbed microhabitat (open circle).


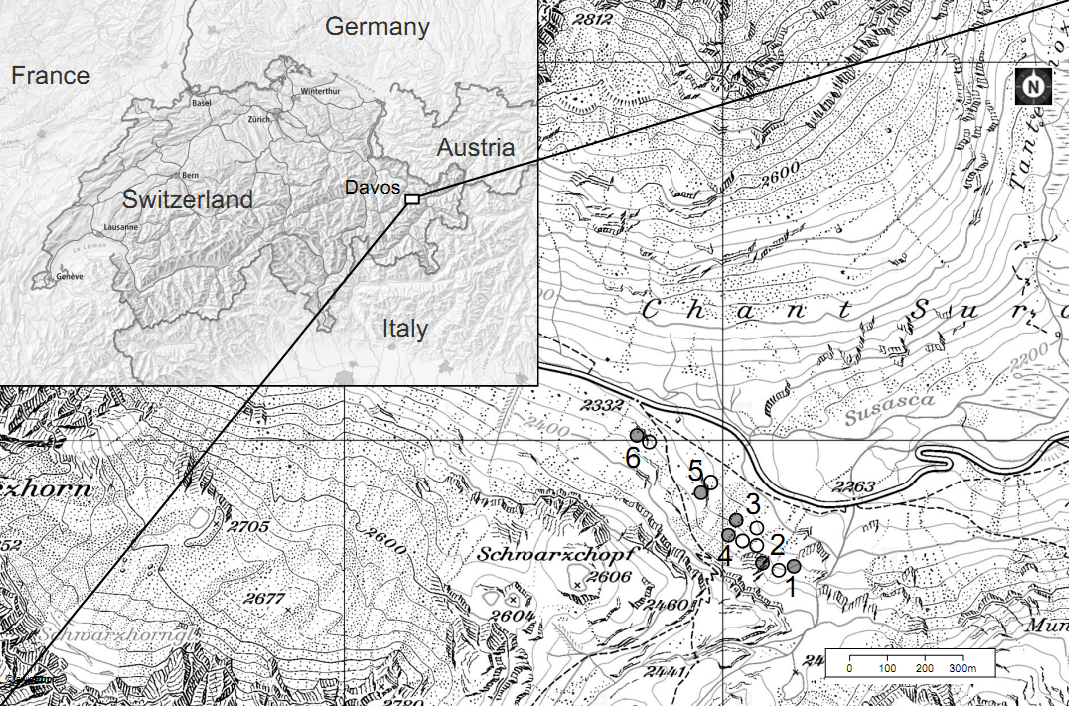

Supplement: S1 Fig — Map of the study area near Davos (Switzerland), and locations of the six pairs of study sites (1–6), each consisting of one early exposed ridge microhabitat (filled circle) and one late exposed snowbed microhabitat (open circle). (DOCX) [file pone.0122395.s002.docx]
