# Supplementary material for: The Response of the Alpine Dwarf Shrub Salix herbacea to Altered Snowmelt Timing: Lessons from a Multi-Site Transplant Experiment
Source: PLoS One. 2015 Apr 20;10(4):e0122395. doi: 10.1371/journal.pone.0122395 (PMC4403918; doi:10.1371/journal.pone.0122395)

**S2 Fig.** **Accumulated growing degree days**. Accumulated growing degree days on ridge- (red) and snowbed sites (blue) in 2012 and 2013. The data were collected on six ridge and six snowbed microhabitat sites used in a reciprocal transplant study established in 2011 in an alpine site near Flüelapass, Switzerland.


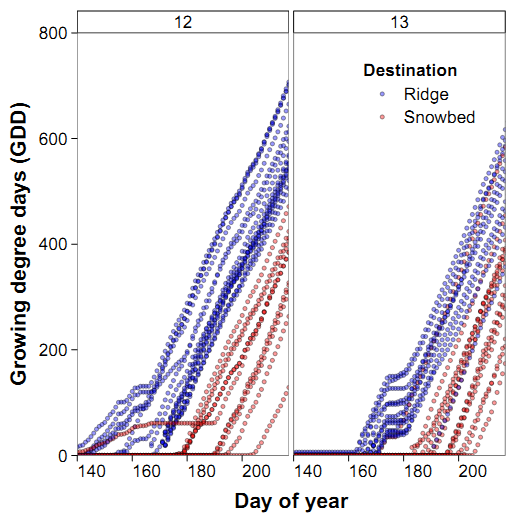

Supplement: S2 Fig — Accumulated growing degree days (GDD, 5°C baseline) on ridge- (red) and snowbed sites (blue) in 2012 and 2013. The data were collected on six ridge and six snowbed microhabitat sites used in a reciprocal transplant study established in 2011 in an alpine site near Flüelapass, Switzerland. (DOCX) [file pone.0122395.s003.docx]
