# Supplementary material for: The Response of the Alpine Dwarf Shrub Salix herbacea to Altered Snowmelt Timing: Lessons from a Multi-Site Transplant Experiment
Source: PLoS One. 2015 Apr 20;10(4):e0122395. doi: 10.1371/journal.pone.0122395 (PMC4403918; doi:10.1371/journal.pone.0122395)
Supplement: S1 Table — Geographical coordinates, mean snowmelt date, mean growing season temperature and accumulated growing degree days (GDD, 5°C baseline) over the growing season of the 6 ridge and 6 snowbed microhabitat sites used in a reciprocal transplant study established in 2011 in an alpine site near Flüelapass, Switzerland. (DOCX) [file pone.0122395.s004.docx]

**S1 Table. Study site characteristics.** Geographical coo­­­rdinates, mean snowmelt date, mean growing season soil temperature and accumulated growing degree days (GDD, 5°C baseline) over the growing season of the 6 ridge and 6 snowbed microhabitat sites used in a reciprocal transplant study established in 2011 in an alpine site near Flüelapass, Switzerland.

| **Site** | **Latitude (°)** | **Longitude (°)** | **Snowmelt day 2012** | **Snowmelt day2013** | **Growing season soil Temp [°C] 2012** | **Growing season soil Temp [°C] 2013** | **GDD 2012** | **GDD 2013** |
| --- | --- | --- | --- | --- | --- | --- | --- | --- |
| 1-Ridge | 46.74016667 | 9.96690556 | 135 | 163 | 8.5 | 11.5 | 1405 | 1226 |
| 2-Ridge | 46.74026944 | 9.96581667 | 167 | 167 | 10.7 | 12.4 | 848 | 526 |
| 3-Ridge | 46.74131389 | 9.96495000 | 136 | 164 | 11.2 | 12.3 | 1084 | 1189 |
| 4-Ridge | 46.74095833 | 9.96466389 | 163 | 170 | 9.6 | 12.9 | 943 | 811 |
| 5-Ridge | 46.74200000 | 9.96375833 | 154 | 164 | 10.4 | 9.8 | 1414 | 1330 |
| 6-Ridge | 46.74340278 | 9.96161667 | 141 | 166 | 12.8 | 8.5 | 654 | 257 |
| 1-Snowbed | 46.74008333 | 9.96637500 | 178 | 187 | 11.0 | 10.3 | 1234 | 1193 |
| 2-Snowbed | 46.74068889 | 9.96563611 | 170 | 180 | 12.7 | 14.1 | 908 | 643 |
| 3-Snowbed | 46.74110833 | 9.96565833 | 198 | 190 | 11.5 | 9.8 | 1117 | 715 |
| 4-Snowbed | 46.74081111 | 9.96515000 | 198 | 195 | 12.8 | 13.4 | 1167 | 606 |
| 5-Snowbed | 46.74218056 | 9.96396389 | 197 | 198 | 9.9 | 9.4 | 1074 | 1039 |
| 6-Snowbed | 46.74323611 | 9.96196944 | 202 | 205 | 14.2 | 13.4 | 815 | 555 |
