# Supplementary material for: The Response of the Alpine Dwarf Shrub Salix herbacea to Altered Snowmelt Timing: Lessons from a Multi-Site Transplant Experiment
Source: PLoS One. 2015 Apr 20;10(4):e0122395. doi: 10.1371/journal.pone.0122395 (PMC4403918; doi:10.1371/journal.pone.0122395)
Supplement: S2 Table — The effects of destination, origin, year and their interactions, on phenology (onset of leaf expansion, onset of flowering, phenological development time to flowering, phenological development time to fruiting) of reciprocally transplanted Salix herbacea turfs. Ratio of flowering and fruiting stems was measured only in 2013, so year was excluded from these models. Initial stem number of 2011 was used as a covariate in the model for stem number (see Methods for details). Log-likelihood ratio tests were used to obtain χ2 test statistic. (DOCX) [file pone.0122395.s005.docx]

**S2 Table. Results for flowering and fruiting.** The effects of destination, origin, year and their interactions, on phenology (onset of leaf expansion, onset of flowering, phenological development time to flowering, phenological development time to fruiting) of reciprocally transplanted *Salix herbacea* turfs. Log-likelihood ratio tests were used to obtain χ^2^ test statistic. We also report delta AIC (dAIC) values for the model comparisons (see Methods for details on the comparisons).

|  | **Onset of leaf expansion** | | | **Onset of flowering** | | | | **Development time to flowering** | | | | | **Onset of fruiting** | | | **Development time to fruiting** | | | |
| --- | --- | --- | --- | --- | --- | --- | --- | --- | --- | --- | --- | --- | --- | --- | --- | --- | --- | --- | --- |
| **Source of Variation** | dAIC | χ**^2^** | ***P*** | | dAIC | χ**^2^** | ***P*** | | dAIC | χ**^2^** | ***P*** | dAIC | | χ**^2^** | ***P*** | | dAIC | χ**^2^** | ***P*** |
| Year | 39.31 | 41.30 | **<0.001** | | 57.09 | 59.09 | **<0.001** | | -1.73 | 0.27 | 0.602 | 8.73 | | 10.73 | **0.001** | | 1.91 | 3.91 | **0.048** |
| Destination | 23.33 | 25.33 | **<0.001** | | 17.99 | 19.99 | **<0.001** | | 7.53 | 9.53 | **0.002** | 20.60 | | 22.60 | **<0.001** | | 1.15 | 3.15 | 0.076 |
| Origin | 2.13 | 2.13 | 0.144 | | -0.69 | 1.31 | 0.252 | | -1.88 | 0.12 | 0.728 | -0.43 | | 1.57 | 0.210 | | -0.65 | 1.35 | 0.246 |
| Destination : Year | 99.98 | 101.99 | **<0.001** | | 74.17 | 76.17 | **<0.001** | | -1.47 | 0.53 | 0.468 | 8.64 | | 10.64 | **0.001** | | 2.17 | 4.17 | **0.041** |
| Origin : Year | 0.61 | 2.61 | 0.107 | | -1.99 | 0.01 | 0.917 | | -0.49 | 1.51 | 0.219 | -1.97 | | 0.03 | 0.864 | | -1.75 | 0.25 | 0.620 |
| Origin : Destination | -1.69 | 0.31 | 0.578 | | -2.00 | <0.01 | 0.986 | | -2.00 | <0.01 | 0.984 | -1.29 | | 0.71 | 0.400 | | -1.34 | 0.66 | 0.417 |
| Origin : Destination : Year | -1.61 | 0.39 | 0.531 | | -1.69 | 0.31 | 0.576 | | -1.19 | 0.81 | 0.369 | -1.98 | | 0.02 | 0.892 | | -1.99 | 0.01 | 0.927 |
| **Random effects** | **-** | **SD** | | |  | **SD** | | |  | **SD** | |  | | **SD** | | |  | **SD** | |
| Turf/Patch/Plot/Site | - | <0.0001 | | | - | 2.6310 | | | - | <0.0001 | | - | | 3.7640 | | | - | 3.1410 | |
| Patch/Plot/Site | - | <0.0001 | | | - | 2.593 | | | - | <0.0001 | | - | | 1.3780 | | | - | 3.6780 | |
| Plot/Site | - | 5.1790 | | | - | 7.2390 | | | - | 3.3340 | | - | | 4.7550 | | | - | 8.9110 | |
| Site | - | <0.0001 | | | - | <0.0001 | | | - | 2.8870 | | - | | <0.0001 | | | - | <0.0001 | |
| Residual | - | 5.2800 | | | - | 5.0790 | | | - | 6.7960 | | - | | 7.3030 | | | - | 7.0760 | |
| **marginal *R*^2^; conditional *R*^2^** | - | 0.779 | 0.879 | | - | 0.743 | 0.923 | | - | 0.343 | 0.613 | - | | 0.712 | 0.833 | | - | 0.210 | 0.737 |
| **AIC (full model)** | 2573.8 | - | - | | 1336.9 | - | - | | 1362.0 | - | - | 673.8 | | - | - | | 704.0 | - | - |
