# Supplementary material for: The Response of the Alpine Dwarf Shrub Salix herbacea to Altered Snowmelt Timing: Lessons from a Multi-Site Transplant Experiment
Source: PLoS One. 2015 Apr 20;10(4):e0122395. doi: 10.1371/journal.pone.0122395 (PMC4403918; doi:10.1371/journal.pone.0122395)
Supplement: S3 Table — The formulas of the full model (1) and the submodels (2–9 or 2–11) are given in the lower part of the table. (DOCX) [file pone.0122395.s006.docx]

**S3 Table. Model comparisons used for the results presented in Table 1of the main manuscript.** The formulas of the full model (1) and the submodels (2-9 or 2-11) are given in the lower part of the table.

| **Source of variation** | **Leaf size,**  **Onset of leaf expansion,**  **Development time to leaf expansion,**  **Leaf damage** | **Stem number** | **Flowering stem ratio** |
| --- | --- | --- | --- |
| No. stems 2011 (N) | - | 11 vs 10 | - |
| Year (Y) | 9 vs 6 | 9 vs 6 | - |
| Sex (S) | **-** | **-** | 9 vs 6 |
| Destination (D) | 8 vs 6 | 8 vs 6 | 8 vs 6 |
| Origin (O) | 7 vs 6 | 7 vs 6 | 7 vs 6 |
| Destination:Year | 5 vs 2 | 5 vs 2 | - |
| Destination:Sex | - | - | 5 vs 2 |
| Origin:Year | 4 vs 2 | 4 vs 2 | - |
| Origin:Sex | - | - | 4 vs 2 |
| Origin:Destination | 3 vs 2 | 3 vs 2 | 3 vs 2 |
| Origin:Destination:Year | 2 vs 1 | 2 vs 1 | - |
| Origin:Destination:Sex | - | - | 2 vs 1 |
| **Models** | (1) Y + D + O + D×Y + O×Y + O×D + O×D×Y | (1) N + Y + D + O + D×Y + O×Y + O×D + O×D×Y | (1) S + D + O + D×S + O×S + O×D + O×D×S |
|  | (2) Y + D + O + D×Y + O×Y + O×D | (2) N + Y + D + O + D×Y + O×Y + O×D | (2) S + D + O + D×S + O×S + O×D |
|  | (3) Y + D + O + D×Y + O×Y | (3) N + Y + D + O + D×Y + O×Y | (3) S + D + O + D×S + O×S |
|  | (4) Y + D + O + D×Y + O×D | (4) N + Y + D + O + D×Y + O×D | (4) S + D + O + D×S + O×D |
|  | (5) Y + D + O + O×Y + O×D | (5) N + Y + D + O + O×Y + O×D | (5) S + D + O + O×S + O×D |
|  | (6) Y + D + O | (6) N + Y + D + O | (6) S + D + O |
|  | (7) Y + D | (7) N + Y + D | (7) S + D |
|  | (8) Y + O | (8) N + Y + O | (8) S + O |
|  | (9) D + O | (9) N + D + O | (9) D + O |
|  |  | (10) N |  |
|  |  | (11) Intercept only |  |
